# Glycerol/NaCl-Regulated Poly(vinyl alcohol)/Sodium Alginate/Graphene Oxide Composite Gels with Low-Temperature Flexibility and a Strain-Dependent Resistance Response

**DOI:** 10.3390/gels12090816

**Published:** 2026-09-06

**Authors:** Jiajun Liu, Fuqiang Chu, Haikuo Zhang, Jilei Chao

**Affiliations:** 1 Faculty of Light Industry, Qilu University of Technology (Shandong Academy of Sciences), Jinan 250353, China; 10431240402@stu.qlu.edu.cn (J.L.); fqchu@qlu.edu.cn (F.C.);; 2 State Key Laboratory of Green Papermaking and Resource Recycling, Faculty of Light Industry, Qilu University of Technology (Shandong Academy of Sciences), Jinan 250353, China

**Keywords:** poly(vinyl alcohol), sodium alginate, graphene oxide, glycerol, antifreezing, low-temperature flexibility, resistive strain sensing

## Abstract

Flexible gel sensors can lose mechanical compliance and electrical stability at low temperature or during solvent loss. A poly(vinyl alcohol) (PVA)/sodium alginate (SA)/graphene oxide (GO) composite gel was prepared by freeze–thaw cycling and post-treated in either aqueous NaCl or a NaCl-containing water/glycerol mixture (1:2, *v*/*v*). The water/glycerol–NaCl-treated gel (F-G/S/P/G) exhibited a maximum tensile stress of 428 ± 27 kPa and an elongation at break of 432 ± 27% at room temperature (*n* = 3), and remained visibly deformable after 24 h at −20 °C. During ambient storage, it retained approximately 89% of its initial mass after 35 days. The cycle-averaged peak ΔR/R_0_ increased from 0.135 at 20% strain to 1.142 at 250% strain, and the 20–60% linear region gave a gauge factor of 1.01 (R^2^ = 0.9988). Three independently prepared sensing elements gave a peak ΔR/R_0_ of 0.710 ± 0.019 at 100% strain, with response and recovery times of 1.22 ± 0.07 and 1.04 ± 0.06 s, respectively. After 500 cycles at 100% strain, the normalized peak response retained 95.1% of its initial value. Overall, F-G/S/P/G combined low-temperature deformability, ambient-storage mass retention, and repeatable resistance sensing.

## 1. Introduction

Flexible wearable electronics require soft sensing materials that can conform to curved, moving surfaces and convert mechanical deformation into measurable electrical signals. Conductive gels are attractive candidates because their solvent-rich polymer networks combine compliance, stretchability, and electrical responsiveness [1,2,3]. Recent reviews have highlighted their use in motion monitoring, health-related sensing, and human–machine interfaces [4,5].

A major limitation of water-containing gels is their susceptibility to freezing and solvent evaporation, both of which can degrade mechanical and sensing performance. Current anti-freezing and anti-drying strategies mainly involve network engineering, solvent replacement, and ionic modification [6,7]. Hydrogen-bond-rich hydrogels and solvent-replaced double-network systems have shown that regulating both the liquid phase and the polymer network can improve subzero flexibility and mass retention [8,9]. Mechanically reinforced double networks, interlocked interfaces, and organohydrogel designs can also preserve deformation-dependent electrical responses under subzero conditions or during prolonged storage [10,11,12].

Multicomponent polymer networks provide a versatile route to balancing mechanical integrity and electrical response. Conductive composite hydrogels, graphene oxide (GO)-modulated hierarchical networks, and ion-containing gels have shown that multiple physical interactions can enhance stress transfer and stabilize strain-dependent signals [13,14,15]. Poly(vinyl alcohol)/sodium alginate (PVA/SA)-based conductive gels, alginate-containing graphene networks, and multiply crosslinked PVA/SA sensors have also been explored for flexible sensing [16,17,18]. Mehrjou et al. incorporated PEG-grafted GO into a PVA/SA semi-interpenetrating network and observed changes in both structure and mechanical behavior [19]. Stretchable SA ionic hydrogel fibers further demonstrate the suitability of alginate-based networks for detecting large strains [20].

Polyols and dissolved salts are widely used to regulate the liquid phase of low-temperature ionic gels. In PVA–NaCl–glycerol and PVA/SA/glycerol systems, glycerol modifies hydrogen bonding, depresses water freezing, and reduces solvent loss, while dissolved salts provide mobile ions and alter the hydration environment [21,22,23]. A microfiber-reinforced PVA hydrogel containing glycerol and NaCl also showed improved ionic conduction and dehydration resistance in flexible bioelectronic applications [24]. Related polyol-containing and ionic hydrogels have combined high stretchability with subzero tolerance and human-motion sensing [25,26,27], and antifreezing strain sensing has been reported for PPy/cellulose-nanofiber-reinforced PVA hydrogels [28]. Excessive plasticization, however, can reduce load-bearing capacity, making glycerol content an important formulation variable.

Direct ink writing (DIW) enables hydrogel inks to be patterned directly on flexible substrates [29,30,31,32,33,34,35]. Here, G/S/P/G2 ink was deposited on PET to form sensing strips, while molded specimens were used for tensile testing where a defined geometry was required. Both routes used the same precursor formulation and the same freeze–thaw and post-treatment sequence.

PVA/SA-based gels and glycerol- or salt-containing organohydrogels have been widely investigated for low-temperature sensing [21,22,23,24,25,26,27,28]. In the present work, the freeze–thawed G/S/P/G2 precursor was kept unchanged while the post-treatment medium was varied between aqueous NaCl and NaCl in a water/glycerol mixture (1:2, *v*/*v*). Mechanical, structural, thermal, storage, electrical, and sensing properties were then compared between the resulting gels. The water/glycerol–NaCl-treated gel showed higher room-temperature strength and elongation, remained deformable after storage at −20 °C, and maintained a repeatable strain-dependent resistance response.

## 2. Results and Discussion

### 2.1. Formulation Screening and Tensile Properties

**Figure 1:**
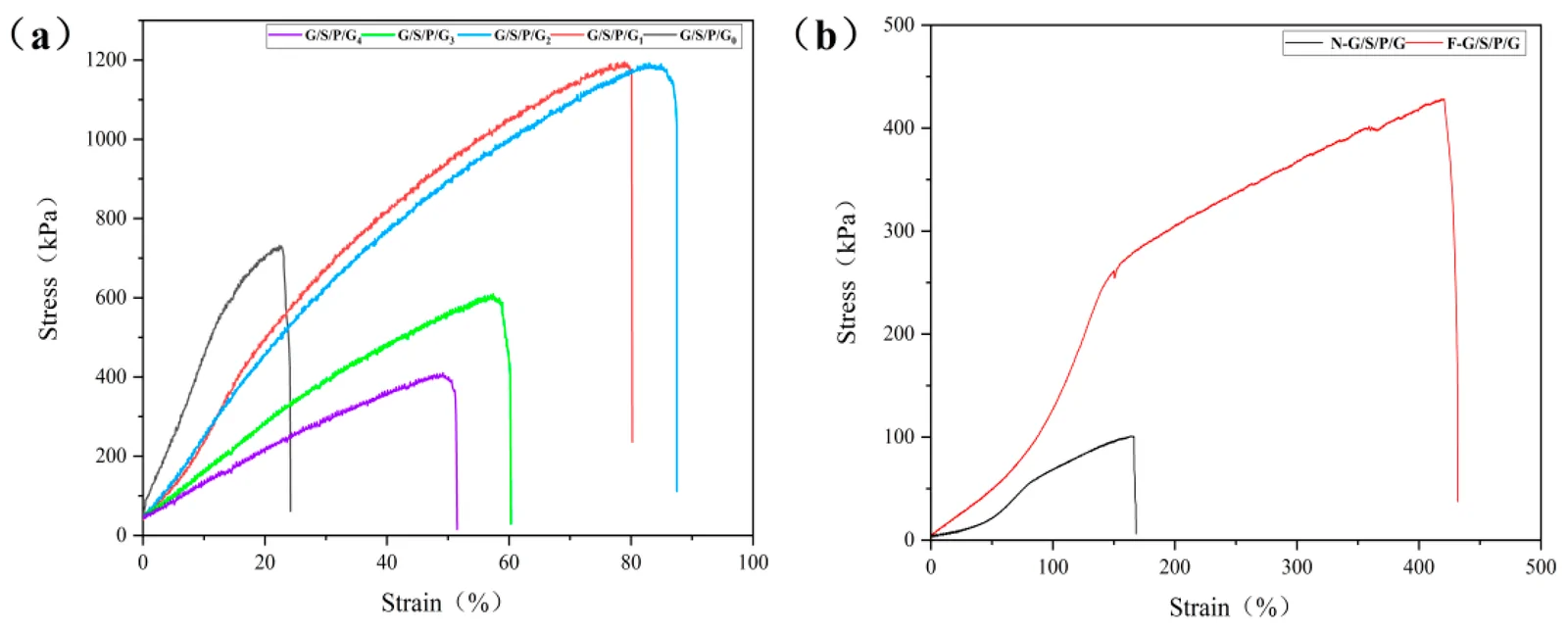
Representative stress–strain curves of the composite materials: (**a**) freeze-dried G/S/P/G0–G4 precursors containing 0–4 g of glycerol per 10 g of precursor ink; (**b**) N-G/S/P/G treated in aqueous NaCl and F-G/S/P/G treated in a NaCl-containing water/glycerol mixture (1:2, *v*/*v*).

**Figure 2:**
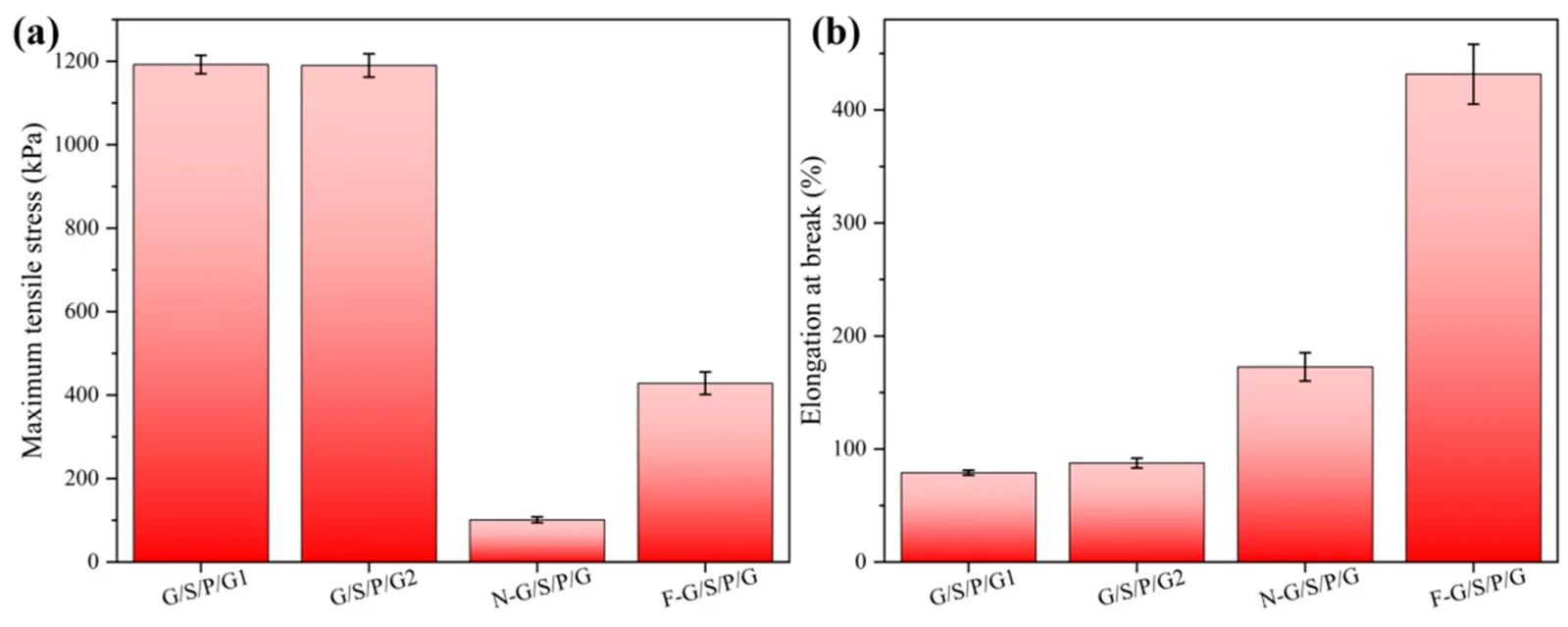
Room-temperature tensile properties of G/S/P/G1, G/S/P/G2, N-G/S/P/G, and F-G/S/P/G: (**a**) maximum tensile stress and (**b**) elongation at break. Data are presented as mean ± SD (*n* = 3 independent specimens).

Figure 1 shows representative stress–strain curves for the freeze-dried G/S/P/G0–G4 precursors and the solvent-treated N-G/S/P/G and F-G/S/P/G gels. Panel (a) shows the glycerol-content screening of the freeze-dried precursors, and panel (b) compares the two post-treatment media. The corresponding mean ± SD values from three independently prepared specimens are shown in Figure 2.

Among the freeze-dried precursors, G/S/P/G1 and G/S/P/G2 gave maximum stresses of 1192 ± 22 and 1190 ± 28 kPa, respectively (*n* = 3; *p* = 0.915), and elongations at break of 80.2 ± 3.8% and 87.5 ± 4.4% (*p* = 0.094). G/S/P/G2 was selected for subsequent post-treatment because it retained the tensile strength of G/S/P/G1 while showing a higher mean elongation at break.

The nonmonotonic effect of glycerol content is consistent with a balance between increased chain mobility and weakened intermolecular interactions. Moderate glycerol addition can reduce local stress concentration, whereas higher glycerol contents increase plasticization and compete for hydrogen-bonding sites, lowering rigidity and load-bearing capacity [21,22,27].

After post-treatment, N-G/S/P/G and F-G/S/P/G showed maximum stresses of 101 ± 7 and 428 ± 27 kPa and elongations at break of 173 ± 13% and 432 ± 27%, respectively (*n* = 3). The differences were significant for both maximum stress (*p* = 0.0013) and elongation at break (*p* = 0.0008). Because the precursor formulation was identical, the post-treatment medium was the main experimental variable underlying these differences.

### 2.2. Freeze-Dried Morphology of the Composite Gel

**Figure 3:**
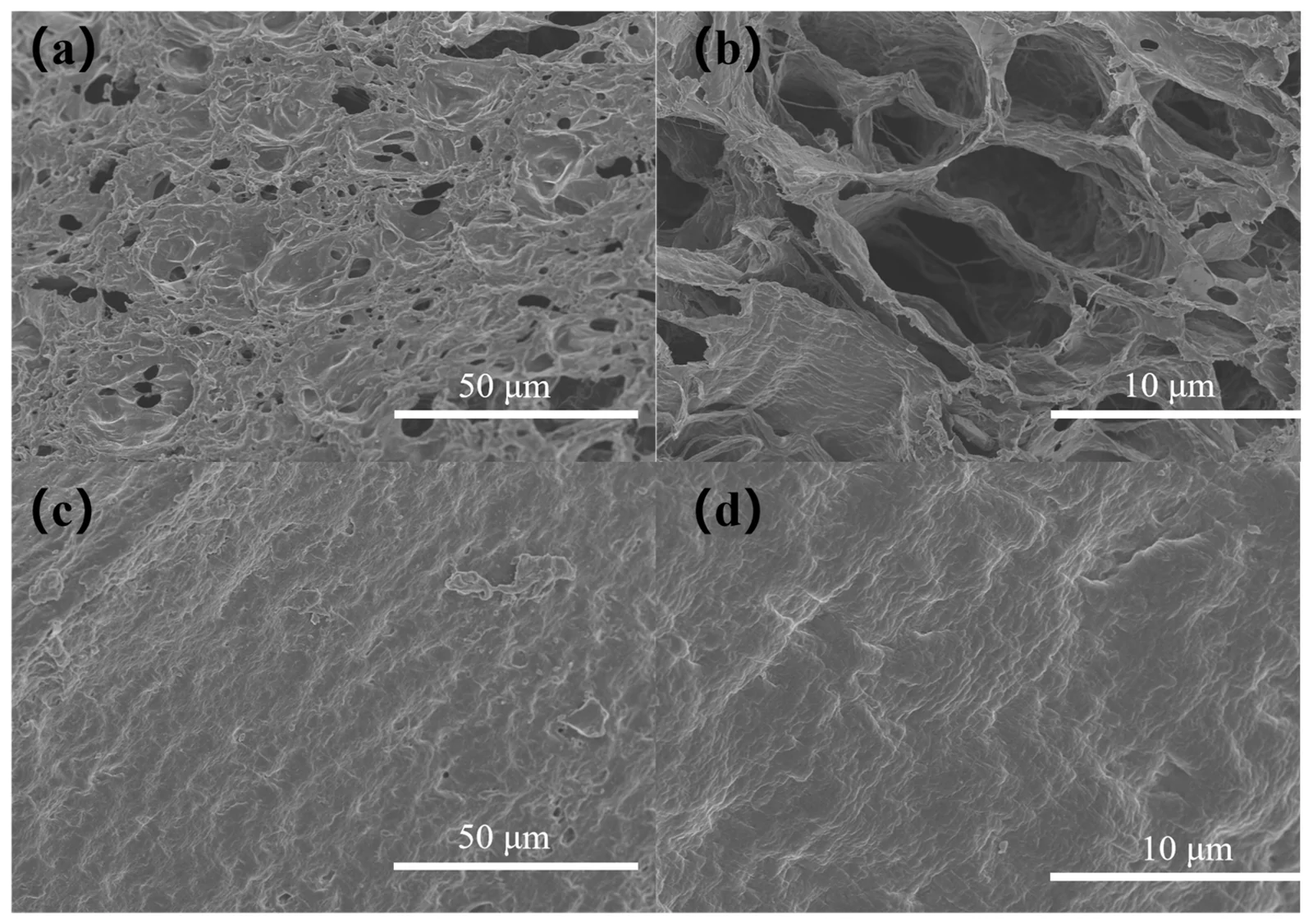
SEM images of freeze-dried F-G/S/P/G: (**a**,**b**) cross-sectional morphology; (**c**,**d**) surface morphology.

Figure 3 shows the freeze-dried surface and cross-sectional morphologies of F-G/S/P/G.

The cross sections (Figure 3a,b) show an interconnected porous architecture with locally layered pore walls, a morphology typical of ice-templated structures formed during freezing and subsequent sublimation. Hydrogen-bonding and interfacial interactions among SA, PVA, and GO can help preserve this network [14,19]. Similar porous structures have been reported in freeze-dried GO/alginate composites [36,37].

The surface images (Figure 3c,d) show a wrinkled, moderately rough texture associated with shrinkage and rearrangement of the PVA/SA/GO network during freezing and drying. Oxygen-containing groups on GO provide additional interaction sites for SA and PVA within the composite.

### 2.3. FTIR Analysis

**Figure 4:**
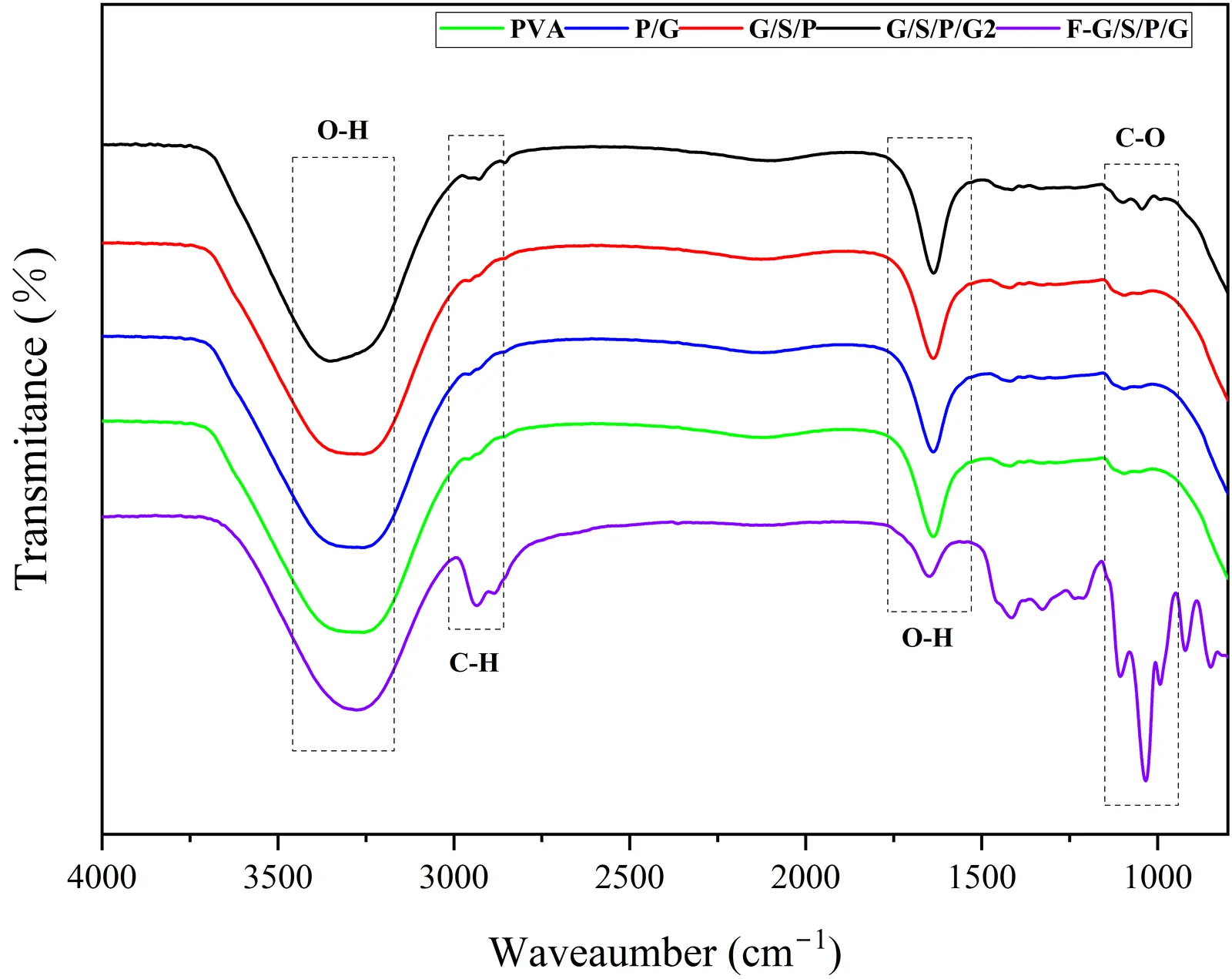
FTIR spectra of PVA, P/G, G/S/P, G/S/P/G2, and F-G/S/P/G.

FTIR spectra of PVA, P/G, G/S/P, G/S/P/G2, and F-G/S/P/G are shown in Figure 4. The broad O–H stretching region (3000–3700 cm^−1^) was further analyzed after baseline correction and normalization. The band center and full width at half maximum (FWHM) were used to compare changes in this region among the samples.

All spectra display a broad band near 3300 cm^−1^, assigned to O–H stretching in PVA, SA, GO, glycerol, and retained water. In the glycerol-containing P/G and G/S/P/G2 samples, changes in the width and apparent intensity of this band reflect the high hydroxyl content of glycerol and changes in the local hydrogen-bonding environment. The hydroxyl- and carboxyl-containing groups on GO can also interact with the polymer components.

After water/glycerol–NaCl treatment, the O–H envelope of F-G/S/P/G becomes slightly shifted and broader, while the C–H stretching band near 2900 cm^−1^ becomes more pronounced because of the methylene groups in glycerol.

The O–H band center changed from approximately 3274.7 cm^−1^ for G/S/P/G2 to 3279.8 cm^−1^ for F-G/S/P/G, while the FWHM increased from approximately 301.8 to 327.1 cm^−1^. The broader envelope indicates a wider distribution of hydrogen-bonding and hydration environments, in agreement with the use of O–H-band analysis to probe water states in hydrogel networks [38]. Because PVA, SA, GO, glycerol, and retained water all contribute in this region, the fitted parameters describe the overall O–H envelope rather than individual hydrogen-bond populations. The derived parameters are summarized in Table 1.

The band near 1640 cm^−1^ contains contributions from water bending and may overlap with vibrations of alginate carboxylate groups. Its lower apparent intensity in F-G/S/P/G suggests a change in retained-water content or state, although the overlapping bands prevent a unique assignment. The bands between 1090 and 1040 cm^−1^ arise mainly from C–O vibrations of PVA, glycerol, and the polysaccharide components, and become more pronounced in the glycerol-containing samples.

### 2.4. XRD Analysis

**Figure 5:**
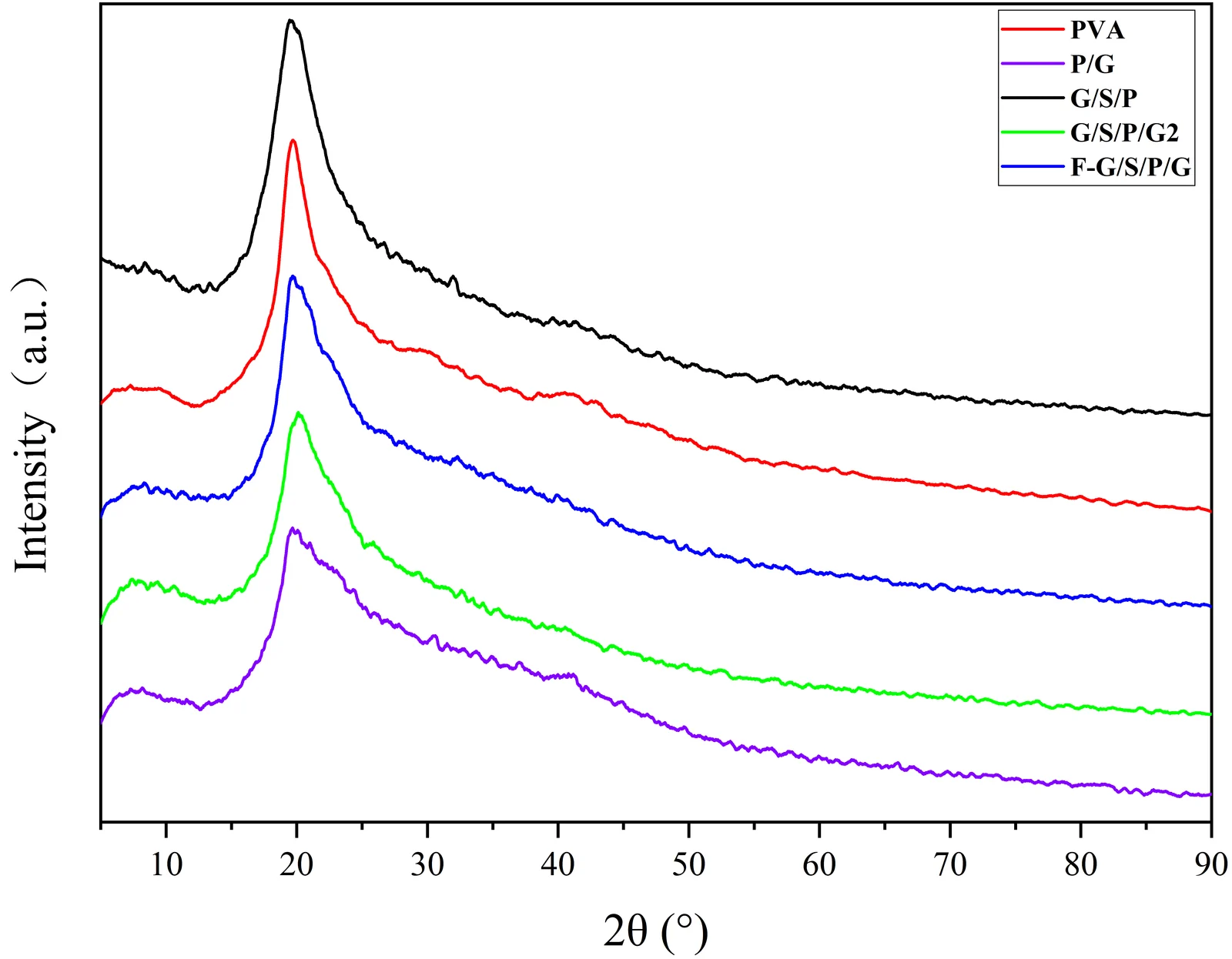
XRD patterns of PVA, P/G, G/S/P, G/S/P/G2, and F-G/S/P/G.

XRD was used to examine the structural ordering of the PVA-containing samples (Figure 5). The patterns were vertically offset to improve readability.

All samples exhibit a broad diffraction feature at 2θ ≈ 19.5°, characteristic of semicrystalline PVA. This feature is comparatively sharper in PVA and G/S/P, indicating greater chain ordering under the sample-preparation conditions used.

The diffraction feature near 19.5° becomes broader and less pronounced in P/G, G/S/P/G2, and F-G/S/P/G. This change is consistent with lower PVA chain ordering in the glycerol-containing samples, as glycerol can interfere with chain packing through competitive hydrogen-bonding interactions [19,21,22].

The principal PVA-related feature remains at nearly the same position in G/S/P/G2 and F-G/S/P/G, suggesting that the water/glycerol–NaCl post-treatment does not substantially change the dominant PVA-related diffraction feature.

### 2.5. DSC Analysis

**Figure 6:**
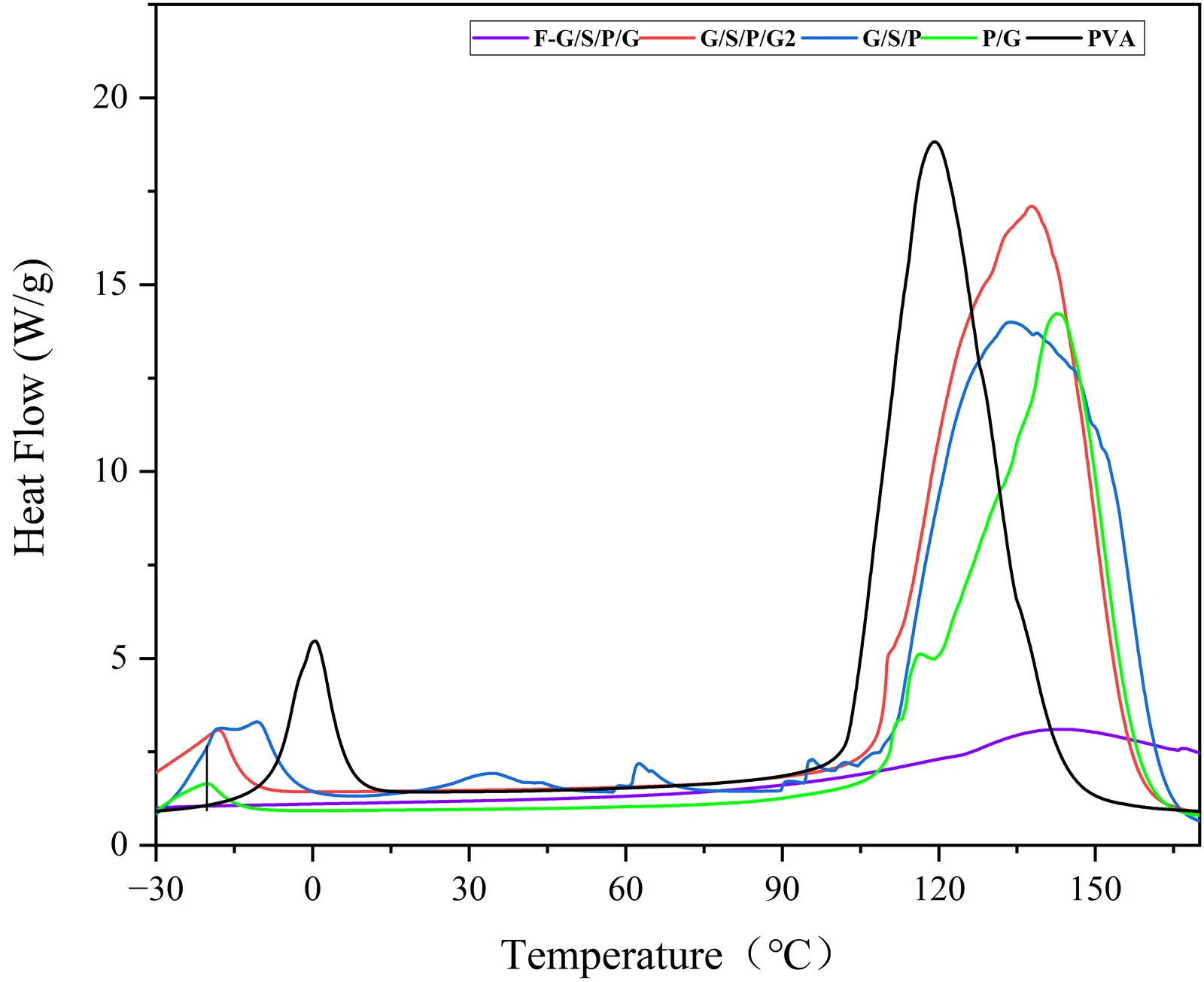
DSC thermograms of PVA, P/G, G/S/P, G/S/P/G2, and F-G/S/P/G gels.

DSC was used to compare solvent-associated thermal transitions among the gel formulations (Figure 6).

The PVA gel exhibits a low-temperature endothermic peak near 0 °C, assigned to the melting of freezable water, and a broader high-temperature endothermic region centered at approximately 119 °C. The latter is associated with the release of bound water and the disruption of polymer–solvent hydrogen bonding.

For P/G, the low-temperature endothermic peak shifts to approximately −20 °C, consistent with glycerol-induced depression of the water-related phase transition [21,22,23]. Similar suppression of water-associated transitions has been reported in glycerol-containing PVA and other antifreezing hydrogel systems [39,40]. The higher-temperature endothermic region of P/G shifts to approximately 142 °C, reflecting changes in polymer–solvent interactions and solvent release.

Analysis of the DSC thermograms gave low-temperature peak temperatures of approximately 0.30 °C for PVA, −20.36 °C for P/G, −10.82 °C for G/S/P, and −18.23 °C for G/S/P/G2. The corresponding transition enthalpies were approximately 203.2, 115.8, 152.5, and 68.0 J g^−1^, respectively. No distinct low-temperature endothermic peak was resolved for F-G/S/P/G within the measured range of −40 to 0 °C, while the broad high-temperature endothermic feature was centered near 143.7 °C. The quantitative DSC parameters are listed in Table 2.

### 2.6. Low-Temperature Deformation and Mass Retention

Low-temperature deformation and room-temperature mass retention were compared for N-G/S/P/G and F-G/S/P/G after the two different post-treatment procedures.

**Figure 7:**
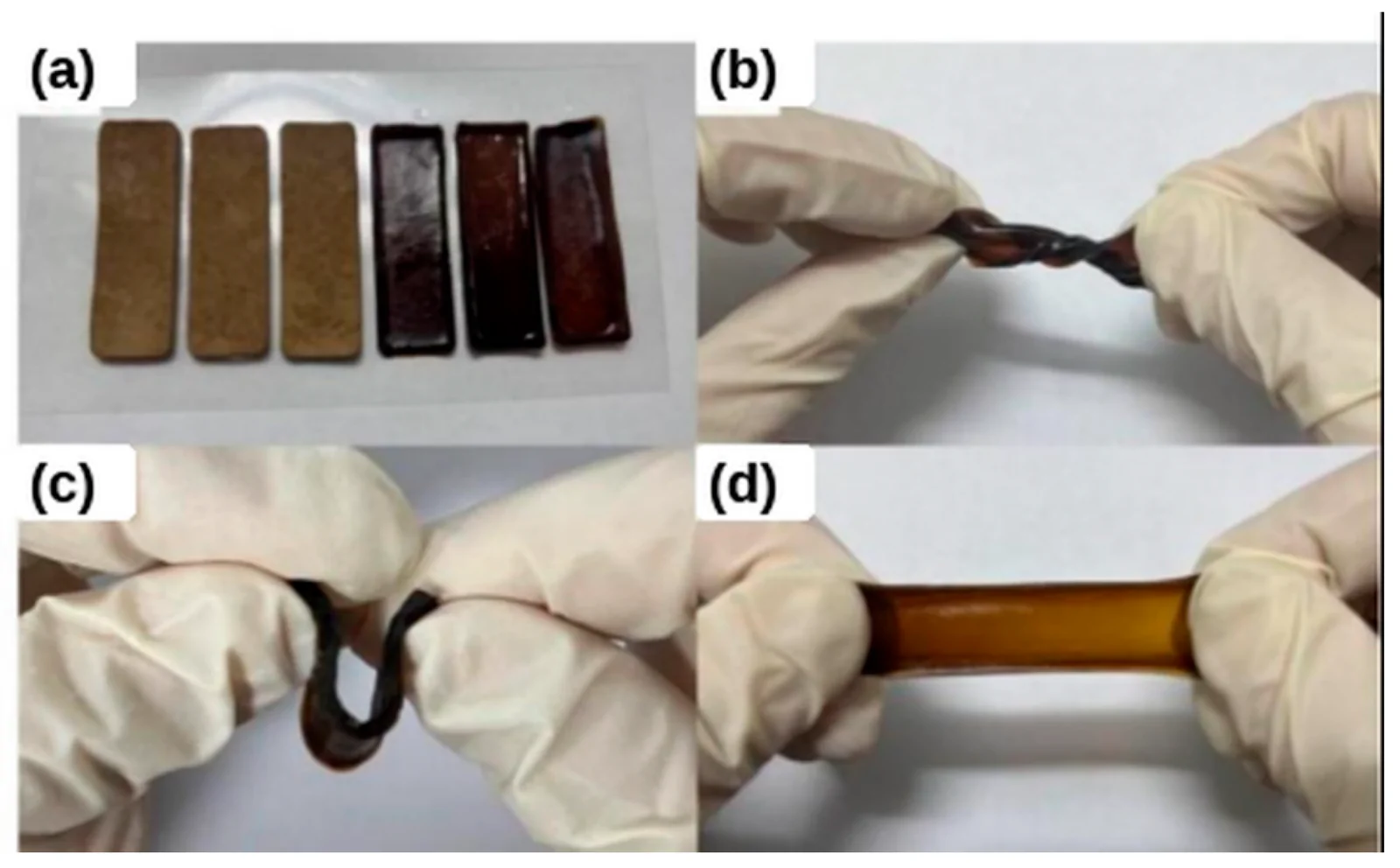
Low-temperature deformation behavior of the composite gels after 24 h at −20 °C: (**a**) appearance of N-G/S/P/G and F-G/S/P/G after low-temperature storage; (**b**) twisting of F-G/S/P/G; (**c**) bending of F-G/S/P/G; (**d**) stretching of F-G/S/P/G in the same low-temperature environment.

After 24 h at −20 °C, N-G/S/P/G appeared frozen and could not be readily deformed, whereas F-G/S/P/G could still be twisted, bent, and stretched without visible fracture (Figure 7). The contrast agrees with the DSC results and with reported glycerol-based antifreezing strategies [8,21,22,39,40], showing that F-G/S/P/G retained greater macroscopic deformability at low temperature.

**Figure 8:**
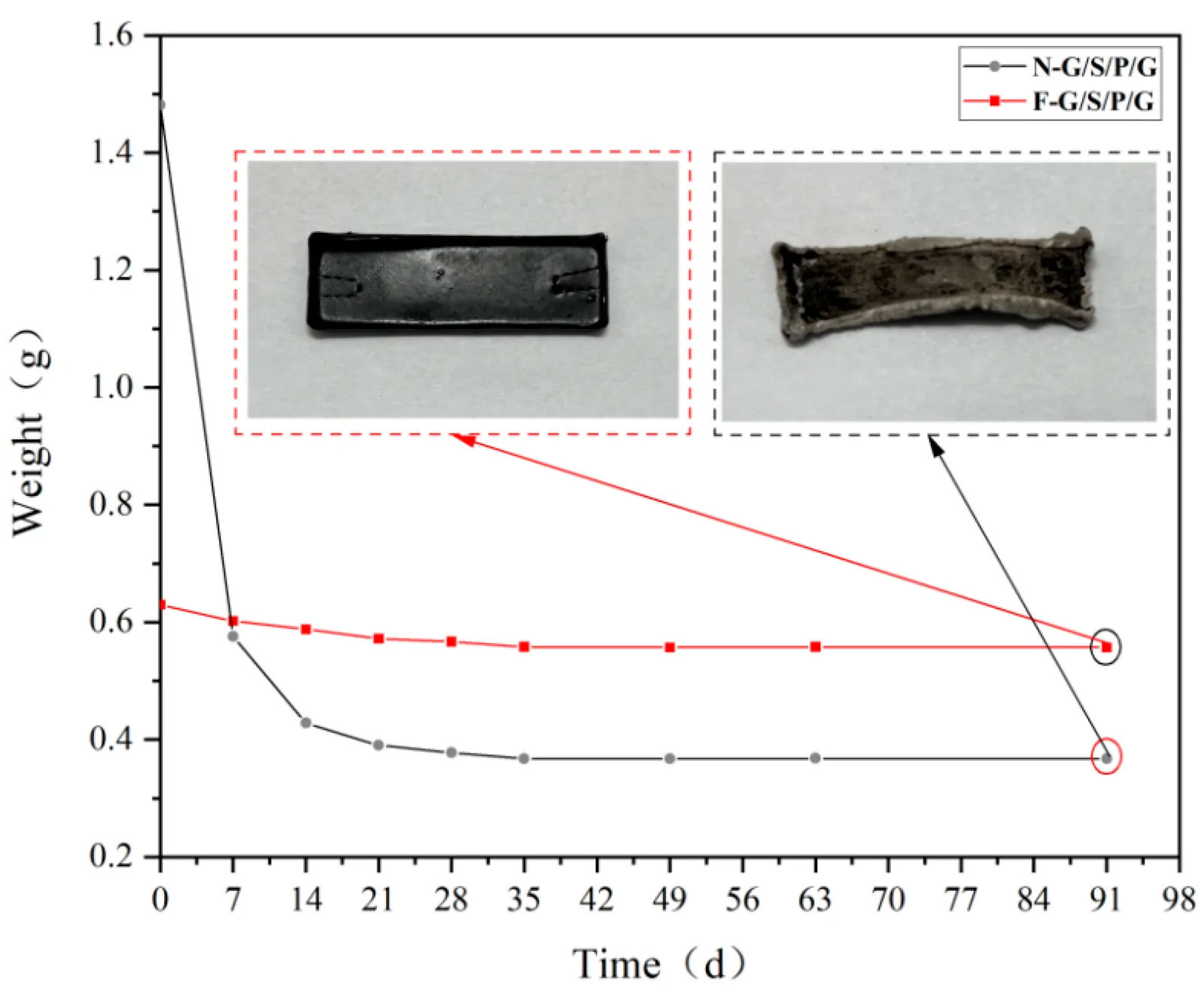
Mass changes in N-G/S/P/G and F-G/S/P/G during room-temperature storage. Retention percentages were calculated relative to the initial mass of each specimen.

Figure 8 shows the mass changes during room-temperature storage. N-G/S/P/G decreased from 1.48 g initially to 0.58 g after 7 days and 0.37 g after 28 days, corresponding to mass retentions of approximately 39.2% and 25.0%, respectively. F-G/S/P/G decreased from 0.63 to 0.56 g after 35 days, retaining approximately 88.9% of its initial mass, and changed only slightly thereafter up to day 91. The higher mass retention of F-G/S/P/G can be related to the low volatility and strong water affinity of glycerol together with polymer–solvent interactions that slow solvent loss [21,23,24,41]. Similar antifreezing and dehydration-resistant behavior has been reported in organohydrogel-based flexible sensors [42]. Because relative humidity was not controlled, the measured mass change represents the net balance of solvent loss and moisture exchange with the ambient environment.

### 2.7. Strain-Sensing Performance and Motion Monitoring

**Figure 9:**
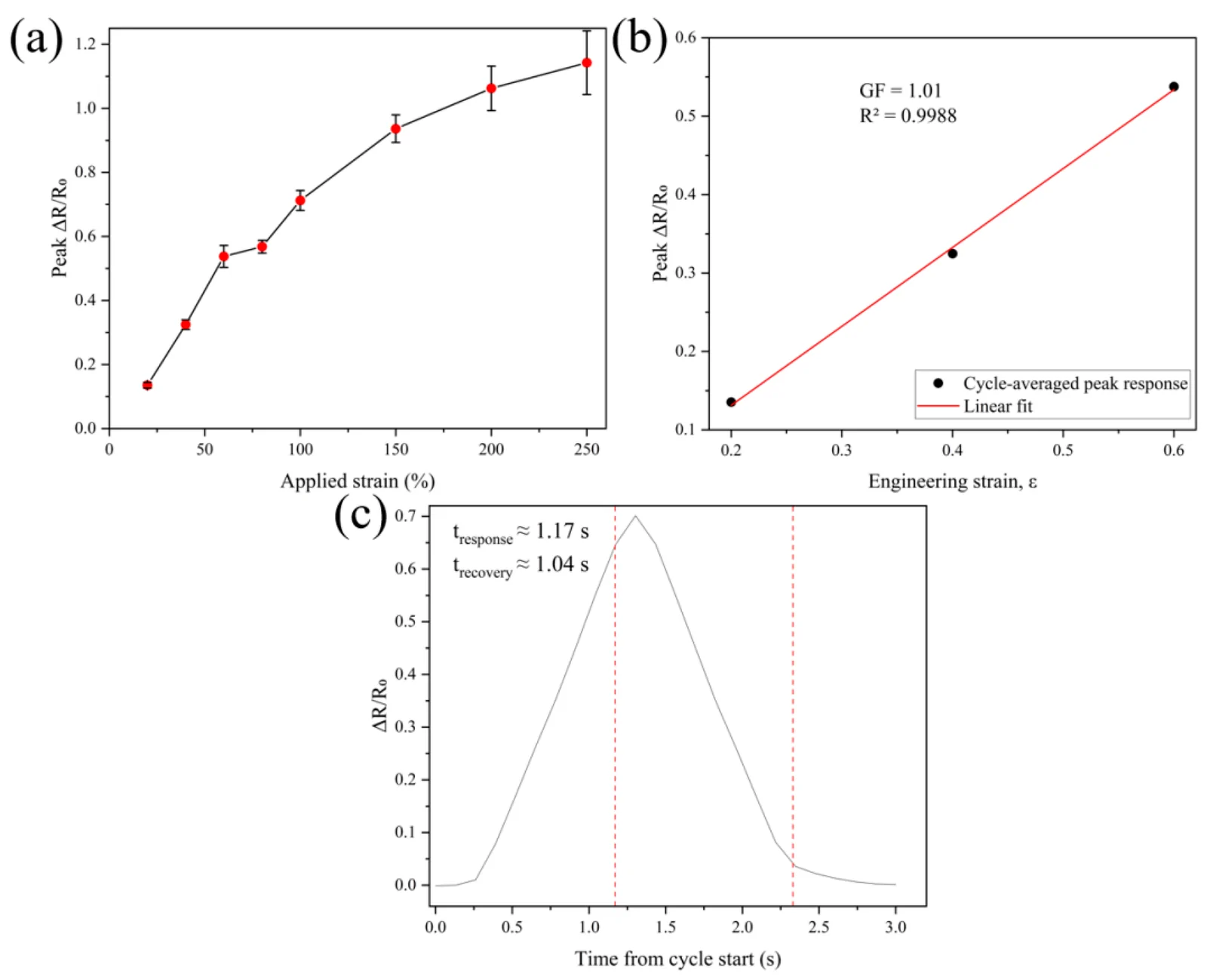
Strain-dependent resistance response of the free-standing F-G/S/P/G sensing element: (**a**) cycle-averaged peak ΔR/R_0_ at 20–250% strain, with the red points representing the cycle-averaged peak values and error bars showing the cycle-to-cycle SD of the same sensing element; (**b**) linear fit over 20–60% strain used to determine GF (GF = 1.01, R^2^ = 0.9988); (**c**) representative loading–unloading cycle at 100% strain, with the red dashed lines indicating the positions used to determine the 90% response and recovery times.

The strain-dependent resistance response of the free-standing F-G/S/P/G sensing element was measured using a digital source meter coupled to a conductive-film testing platform (Figure 9).

The time-resolved resistance data were analyzed using ΔR/R_0_ = (R − R_0_)/R_0_, with R_0_ defined as the unloaded baseline immediately before each loading cycle. The cycle-averaged peak ΔR/R_0_ increased from 0.135 ± 0.009 at 20% strain to 1.142 ± 0.099 at 250% strain. The error bars in Figure 9a represent cycle-to-cycle variation in the same sensing element. Over the initial 20–60% strain range, the peak response was linear with strain, giving a GF of 1.01 (R^2^ = 0.9988).

**Figure 10:**
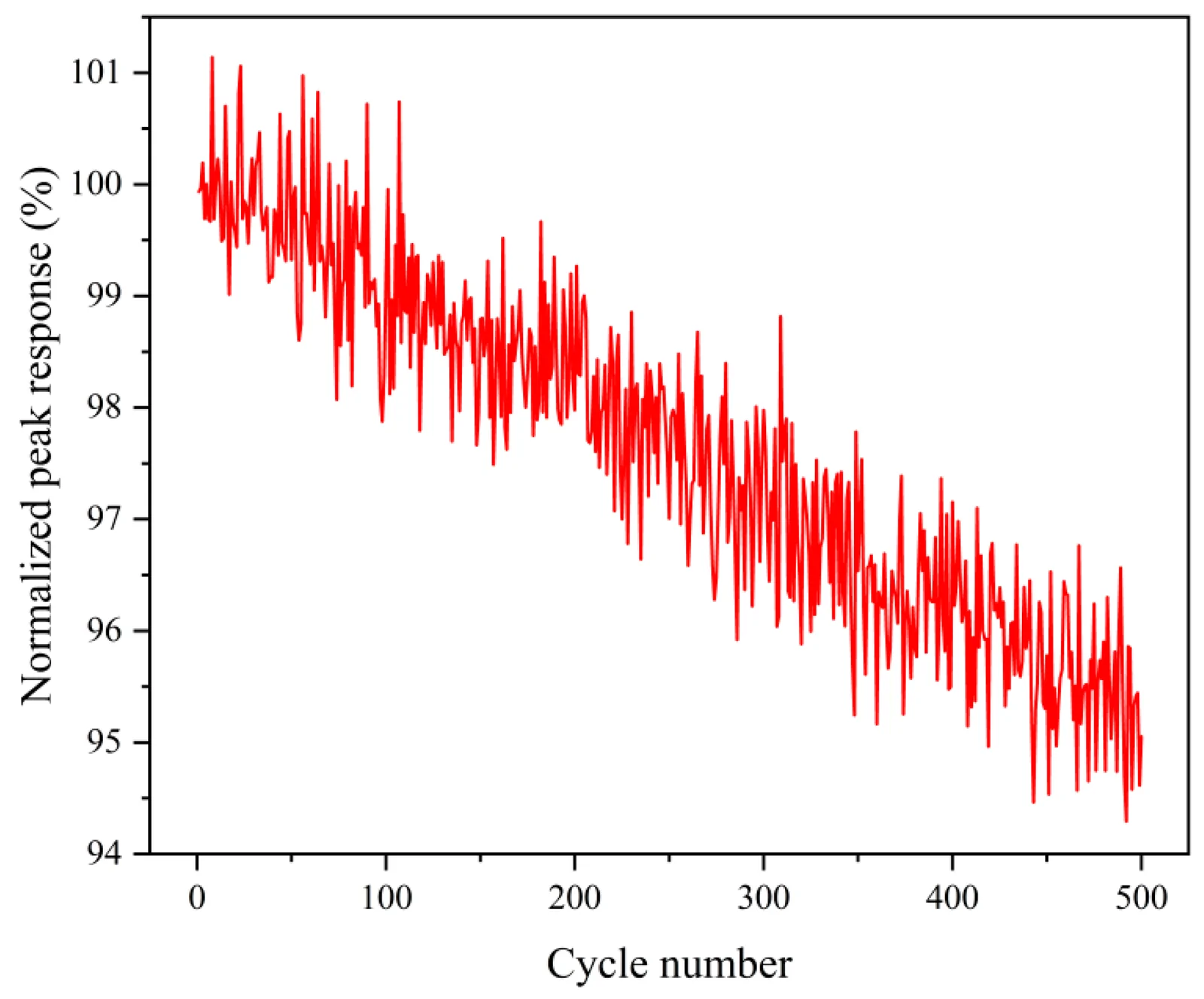
Cycling stability of the F-G/S/P/G sensing element over 500 loading–unloading cycles at 100% strain. The response is normalized to the mean peak response of the first 10 cycles.

**Figure 11:**
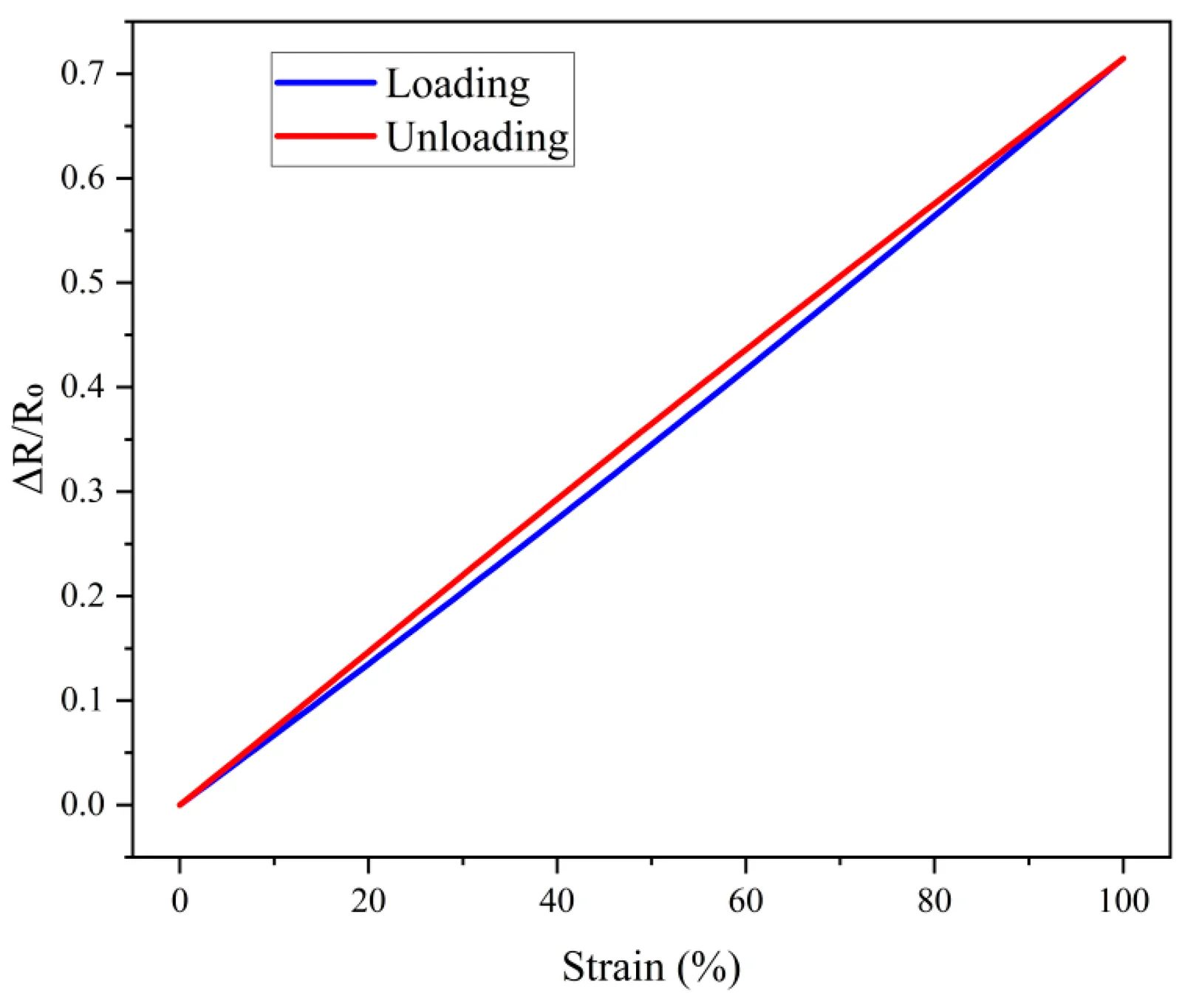
Loading and unloading ΔR/R_0_–strain curves of F-G/S/P/G during a continuous 0 → 100% → 0 strain cycle.

Three independently prepared F-G/S/P/G sensing elements were tested at 100% strain. The peak ΔR/R_0_ was 0.710 ± 0.019, with response and recovery times of 1.22 ± 0.07 and 1.04 ± 0.06 s, respectively (*n* = 3; Table 3). During 500 loading–unloading cycles at 100% strain, the normalized peak response retained 95.1% of its initial value and the unloaded baseline drifted by 2.3% (Figure 10). A continuous 0 → 100% → 0 strain sweep gave a hysteresis ratio of 3.6% (Figure 11).

For motion monitoring, the sensing element was attached to the outer surface of an insulating glove or garment. Conductive adhesive tape provided electrical contact, and insulating tape immobilized the electrode regions while the central gel region remained free to deform. Tests were performed at room temperature and after 24 h of equilibration in an environment maintained at −20 ± 2 °C.

**Figure 12:**
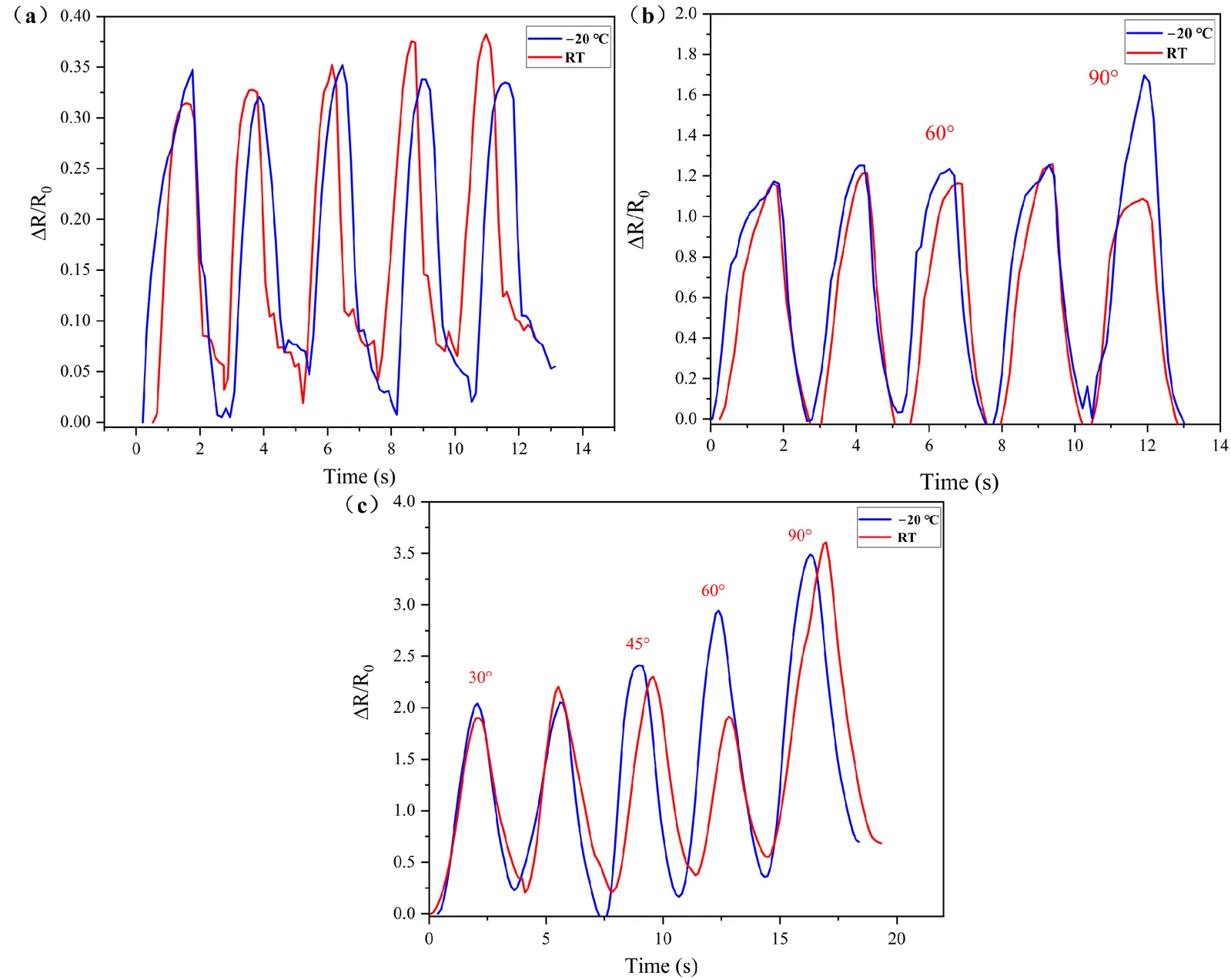
Resistance responses of a free-standing F-G/S/P/G sensing element mounted on the outer surface of an insulating glove or garment at room temperature and −20 ± 2 °C: (**a**) finger; (**b**) wrist; (**c**) elbow.

During finger bending (Figure 12a), ΔR/R_0_ increased during flexion and decreased as the finger returned to its initial position. Similar periodic responses were recorded for wrist and elbow bending at both room temperature and −20 ± 2 °C (Figure 12b,c), indicating that the externally mounted sensing element followed repeated joint motion under both conditions.

At room temperature, the apparent conductivities of N-G/S/P/G and F-G/S/P/G were 0.733 ± 0.028 and 0.518 ± 0.019 mS cm^−1^, respectively (*n* = 3; *p* = 0.0008; Table 4). The lower conductivity of F-G/S/P/G is compatible with slower ion transport in the more viscous glycerol-rich liquid phase. Its strain response therefore arises from deformation of the ionic conduction pathway and specimen geometry rather than from a higher zero-strain conductivity.

### 2.8. Comparison with Representative Antifreezing Hydrogel Systems

Table 5 places F-G/S/P/G alongside representative antifreezing hydrogel strain sensors. Its tensile strength and elongation fall within the range reported for soft antifreezing sensing gels, while it remains visibly deformable after 24 h at −20 °C and retains 95.1% of its initial peak response after 500 cycles. A distinguishing feature of the present comparison is that N-G/S/P/G and F-G/S/P/G were prepared from the same G/S/P/G2 precursor, so the effect of the post-treatment medium can be assessed without changing the precursor network.

GF for F-G/S/P/G was calculated from the slope of the linear response over 20–60% strain (R^2^ = 0.9988). Tensile values for this work are mean ± SD from three independent specimens (*n* = 3).

## 3. Conclusions

A PVA/SA/GO composite gel was prepared by freeze–thaw cycling and treated with either aqueous NaCl or NaCl in a water/glycerol mixture (1:2, *v*/*v*). With the precursor composition fixed, changing the post-treatment medium produced clear differences in tensile behavior, low-temperature deformability, solvent-associated thermal response, and electrical properties.

F-G/S/P/G reached a maximum engineering stress of 428 ± 27 kPa and an elongation at break of 432 ± 27% at room temperature (*n* = 3), remained visibly deformable after 24 h at −20 °C, and retained approximately 89% of its initial mass after 35 days of ambient storage. The 20–60% strain region gave a GF of 1.01 (R^2^ = 0.9988). Three independently prepared sensing elements gave a peak ΔR/R_0_ of 0.710 ± 0.019 at 100% strain, with response and recovery times of 1.22 ± 0.07 and 1.04 ± 0.06 s. After 500 cycles, the normalized peak response retained 95.1% of its initial value, with a measured hysteresis ratio of 3.6%.

G/S/P/G2 ink was also deposited on PET by DIW and converted to free-standing sensing strips through the same freeze–thaw and post-treatment sequence. The strips tracked joint bending when mounted on the outer surface of a glove or garment at room temperature and −20 ± 2 °C. These combined mechanical, environmental, and sensing characteristics are relevant to flexible strain sensing in cold environments.

## 4. Materials and Methods

### 4.1. Materials

Nanographite powder (D_50_ < 400 nm), sodium alginate (SA; supplier-reported viscosity of 5000 mPa·s), glycerol (99%), and poly(vinyl alcohol) (PVA, type 1799) were purchased from Shanghai Macklin Biochemical Co., Ltd. (Shanghai, China). Sodium chloride (NaCl, 99.5%) was obtained from Shanghai Aladdin Biochemical Technology Co., Ltd. (Shanghai, China). Polyethylene terephthalate (PET) substrates were supplied by Beijing Daxiang Plastic Products Factory (Beijing, China). Sulfuric acid (H_2_SO_4_, 98%, AR), hydrogen peroxide solution (H_2_O_2_, 30%, AR), and potassium permanganate (KMnO_4_, AR) were purchased from Sinopharm Chemical Reagent Co., Ltd. (Shanghai, China). All chemicals were used as received unless otherwise stated.

### 4.2. Methods

#### 4.2.1. Preparation of Composite Gels and Sensing Elements

GO dispersion was prepared by a modified Hummers method [43]. SA was added at 10 wt% relative to GO to 10 mL of the GO dispersion, followed by ultrasonication for 0.5 h and mechanical stirring for 20 h to obtain the GO/SA ink. A 10 wt% PVA solution was prepared by allowing 10 g of PVA to swell in 90 g of deionized water at 35 °C for 12 h, followed by heating at 95 °C for 2–3 h until complete dissolution. The PVA solution (7 g) and GO/SA ink (3 g) were then mixed for 3 h and vacuum-degassed for 3 h to produce the G/S/P precursor ink.

A series of precursor inks containing 0, 1, 2, 3, or 4 g of glycerol per 10 g of G/S/P ink was prepared and designated G/S/P/G0, G/S/P/G1, G/S/P/G2, G/S/P/G3, and G/S/P/G4, respectively. Each mixture was stirred for 3 h and vacuum-degassed for a further 3 h. For gel formation, 2 mL of ink was transferred to a rectangular polytetrafluoroethylene mold (2 cm × 1 cm × 1 cm) and subjected to three freeze–thaw cycles, each consisting of freezing at −24 °C for 8 h and thawing at room temperature for 4 h. G/S/P/G2 was used for subsequent post-treatment and sensing tests. The freeze–thawed gels were immersed for 4 h in either aqueous NaCl or a water/glycerol mixture (1:2, *v*/*v*) containing NaCl. For each 100 mL of total initial treatment medium, 11.69 g NaCl was added, corresponding to a nominal concentration of 2.0 mol L^−1^ based on the total initial solvent volume. After treatment, both surfaces of the gels were rinsed with deionized water. The formulations and post-treatment conditions are summarized in Table 6.

The glycerol amount refers to the mass added per 10 g of G/S/P precursor ink, and the NaCl concentration is based on the total initial treatment-medium volume. The equilibrium glycerol and NaCl contents after immersion and rinsing were not measured.

**Figure 13:**
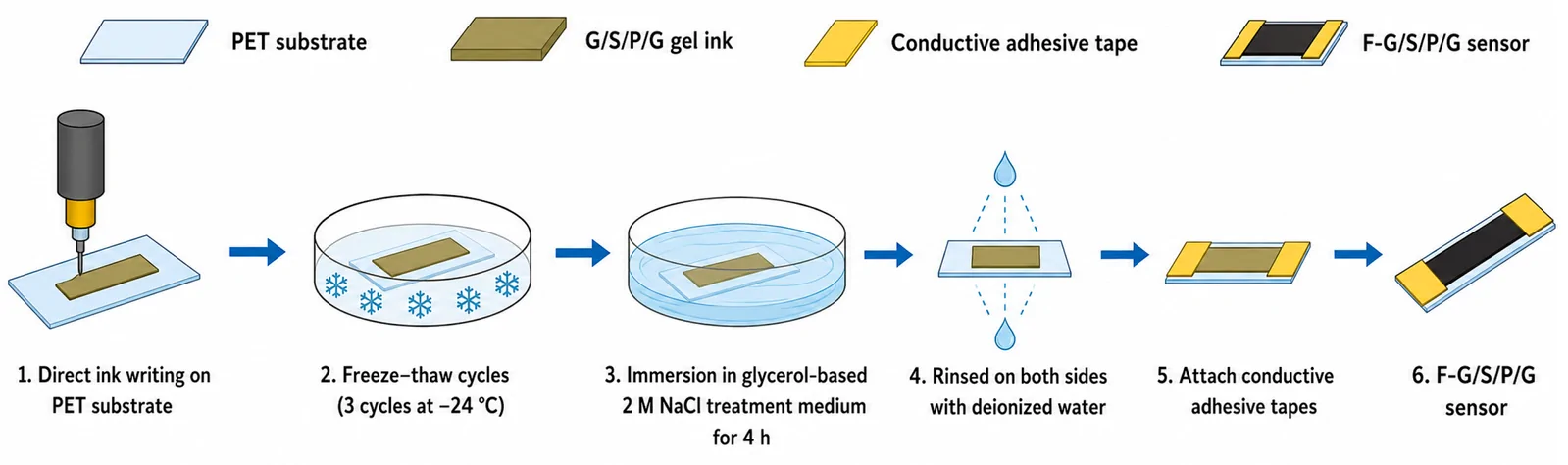
Schematic of composite-gel preparation, direct-ink-writing deposition on PET, freeze–thaw cycling, NaCl-containing post-treatment, rinsing, electrode attachment, and preparation of the strain-responsive sensing element.The colors correspond to the PET substrate, G/S/P/G ink, conductive adhesive tape, and F-G/S/P/G sensor, as indicated in the figure legend.

For DIW, G/S/P/G2 ink was loaded into a 30 mL cartridge and deposited directly onto PET at 10 psi using a printing speed of 4 mm s^−1^ and a 300 μm nozzle. The printed strips underwent the same freeze–thaw and water/glycerol–NaCl treatment procedures and were then peeled from the PET substrate. Conductive adhesive tape was attached to both ends as electrical contacts [34,35]. DIW was used to prepare patterned sensing strips, while molded specimens were used for tensile testing with a defined geometry. The fabrication procedure is shown in Figure 13.

Sample abbreviations are listed in Table 7. The prefixes N and F denote aqueous-NaCl and water/glycerol–NaCl post-treatment, respectively.

#### 4.2.2. Tensile Tests

Tensile tests were performed on G/S/P/G0–G4 and on the solvent-treated N-G/S/P/G and F-G/S/P/G gels using an XLW-EC electronic tensile tester (Jinan Labthink Electromechanical Technology Co., Ltd., Jinan, China). Measurements were conducted at 23 °C and 50% RH with a crosshead speed of 50 mm min^−1^. The freeze-dried G/S/P/G0–G4 strips had a nominal gauge length of approximately 40 mm, a width of 10 mm, and a thickness of approximately 1 mm. The solvent-treated N-G/S/P/G and F-G/S/P/G gels had a nominal gauge length of approximately 20 mm, a width of 10 mm, and a thickness of approximately 10 mm. Engineering stress was calculated from the corresponding initial cross-sectional area, and engineering strain was determined from displacement relative to the initial gauge length.

For G/S/P/G1, G/S/P/G2, N-G/S/P/G, and F-G/S/P/G, three independently prepared specimens were tested under the same room-temperature conditions and the results were reported as mean ± SD. Pairwise comparisons of tensile properties were performed using Welch’s two-sided t-test, with *p* < 0.05 considered statistically significant. Quantitative tensile testing at −20 °C was not performed because the XLW-EC setup did not provide a controlled subzero test environment.

#### 4.2.3. Scanning Electron Microscopy (SEM)

Before SEM analysis, F-G/S/P/G was freeze-dried. Surface and cross-sectional morphologies were observed using a Hitachi Regulus 8220 field-emission scanning electron microscope operated at 5.0 kV. Images were acquired at working distances of approximately 8.4–8.9 mm, with magnifications corresponding to the 50 and 10 μm scale bars shown.

#### 4.2.4. X-Ray Diffraction (XRD)

XRD patterns of PVA, P/G, G/S/P, G/S/P/G2, and F-G/S/P/G were collected using a Rigaku SmartLab diffractometer with Cu Kα radiation (λKα1 = 1.540593 Å) operated at 40 kV and 40 mA. Measurements were performed in continuous-scan mode over 5–90° (2θ), with a step interval of 0.02° and a scan rate of 20° min^−1^.

#### 4.2.5. Fourier Transform Infrared Spectroscopy (FTIR)

FTIR spectra of PVA, P/G, G/S/P, G/S/P/G2, and F-G/S/P/G were acquired using a Bruker ALPHA spectrometer over 4000–400 cm^−1^. A pellet of dried KBr was measured as the background. Each sample was mixed with dried KBr at an approximate mass ratio of 1:100, ground thoroughly, and pressed into a pellet. For analysis of the broad O–H stretching region (3000–3700 cm^−1^), the spectra were baseline-corrected and normalized to the O–H-band maximum; the band center and FWHM were then determined for comparative analysis.

#### 4.2.6. Differential Scanning Calorimetry (DSC)

DSC measurements were performed on PVA, P/G, G/S/P, G/S/P/G2, and F-G/S/P/G using sample masses of approximately 6.5, 6.0, 8.0, 8.0, and 5.8 mg, respectively. Each sample was sealed in an aluminum crucible, cooled to −40 °C, heated to 175 °C at 10 °C min^−1^, held for 10 min, and then cooled back to −40 °C at the same rate. One thermogram was obtained for each formulation. Peak temperatures were read from the thermograms, and low-temperature transition enthalpies were determined by baseline integration where a distinct peak could be reliably identified.

#### 4.2.7. Low-Temperature Deformation and Mass-Retention Tests

N-G/S/P/G and F-G/S/P/G were stored at −20 °C for 24 h and then twisted, bent, and stretched in the same low-temperature environment. For mass-retention measurements, one specimen of each formulation (*n* = 1) was kept in an open Petri dish at room temperature under uncontrolled ambient humidity and followed for 91 days. Mass retention was calculated as *m*_t_/*m*_0_ × 100%, where *m*_0_ is the initial mass and *m*_t_ is the mass at storage time *t*.

#### 4.2.8. Resistance-Response and Motion-Monitoring Tests

The resistance response of a free-standing F-G/S/P/G sensing element was measured using a conductive-film testing platform connected to a Keithley 2400 digital source meter. Measurements were performed in direct resistance mode with an integration time of 2 power-line cycles (NPLC = 2), autozero enabled, and digital filtering disabled. Data were recorded at intervals of approximately 0.13 s. Resistance responses were analyzed at 20, 40, 60, 80, 100, 150, 200, and 250% strain. For each loading cycle, *R*_0_ was defined as the unloaded baseline immediately before loading, and *ΔR/R*_0_ was calculated as (*R* − *R*_0_)/*R*_0_. Peak *ΔR*/*R*_0_ values were averaged over the available cycles at each strain. *GF* was obtained from the slope of the linear fit of peak *ΔR*/*R*_0_ versus engineering strain over 20–60% strain. Response and recovery times were determined using 90% of the total signal change as the criterion.

(1) $$GF=\frac{d(\Delta R/R_{0})}{d\varepsilon }$$

Conductive adhesive tape established electrical contact at both ends of the gel, and insulating tape secured the electrode regions while the central sensing region remained free to deform. For low-temperature motion monitoring, the sensing element, wires, and insulating glove or garment were precooled at −20 °C for 24 h and tested in an environment maintained at −20 ± 2 °C. The sensing element was mounted on the outer surface of the glove or garment and did not directly contact the skin.

Three independently prepared F-G/S/P/G sensing elements were tested at 100% strain to evaluate sensor-to-sensor repeatability. A separate sensing element was subjected to 500 loading–unloading cycles at 100% strain. Response retention was calculated from the mean peak response of cycles 491–500 relative to cycles 1–10, and baseline drift was calculated from the corresponding unloaded resistance values. Hysteresis was evaluated during a continuous 0 → 100% → 0 strain sweep as the area between the loading and unloading curves divided by the area under the loading curve. Apparent conductivity was measured at room temperature for three independent N-G/S/P/G and three independent F-G/S/P/G specimens and calculated as *σ* = *L*/(*RA*), where L is the electrode separation and A is the specimen cross-sectional area. Apparent-conductivity results are reported as mean ± SD, and the two treatment groups were compared using Welch’s two-sided t-test, with *p* < 0.05 considered statistically significant. Because a two-terminal geometry was used, these measurements are reported as apparent conductivity.

## Figures and Tables

**Table 1:** Semi-quantitative parameters of the O–H stretching region after baseline correction and normalization.

Sample	O–H Band Center (cm^−1^)	FWHM (cm^−1^)
PVA	3304.3	375.2
P/G	3275.7	300.8
G/S/P	3329.7	296.4
G/S/P/G2	3274.7	301.8
F-G/S/P/G	3279.8	327.1

**Table 2:** Quantitative DSC parameters obtained from the thermograms.

Sample	Low-Temperature Tpeak (°C)	Low-Temperature ΔH (J g^−1^)	High-Temperature Tpeak (°C)
PVA	0.30	203.2	119.18
P/G	−20.36	115.8	142.78
G/S/P	−10.82	152.5	133.88
G/S/P/G2	−18.23	68.0	137.80
F-G/S/P/G	ND	ND	143.71

**Table 3:** Repeatability of three independently prepared F-G/S/P/G sensing elements at 100% strain. Data are presented as mean ± SD (*n* = 3).

Independent Sensor	Peak ΔR/R_0_ (100%)	Response Time (s)	Recovery Time (s)
1	0.690	1.15	0.98
2	0.711	1.22	1.04
3	0.728	1.28	1.10
Mean ± SD	0.710 ± 0.019	1.22 ± 0.07	1.04 ± 0.06

**Table 4:** Room-temperature apparent conductivity of N-G/S/P/G and F-G/S/P/G. Data are presented as mean ± SD (*n* = 3 independent specimens).

Sample	Apparent Conductivity (mS cm^−1^)	*n*
N-G/S/P/G	0.733 ± 0.028	3
F-G/S/P/G	0.518 ± 0.019	3

**Table 5:** Comparison with representative antifreezing hydrogel strain-sensing systems.

System	Tensile Strength	Elongation at Break	Low-Temperature Condition	GF	Cycles	Ref.
PVA/SA/glycerol + NaCl	1.43 MPa	558%	Anti-freezing behavior reported	—	—	[22]
PVA/PEDOT/PAA/glycerol	~3.6 MPa	—	Responsive at −25 °C	—	—	[25]
PAM/SA/LiCl	0.11 MPa	2100%	Conductive/stretchable at −30 °C	17.45	—	[26]
PVA/EG/MgCl_2_	1.10 MPa	442.3%	Flexible/stable at −20 °C	0.725	50	[27]
PPy/CNF/PVA/glycerol	~0.65 MPa	~301%	Operable at −18 °C	2.84	—	[28]
F-G/S/P/G (this work)	0.428 ± 0.027 MPa	432 ± 27%	Visibly deformable after 24 h at −20 °C	1.01†	500	This work

**Table 6:** Formulations and treatment conditions of the composite samples.

Sample	Base Formulation	Added Glycerol	Post-Treatment	Purpose/Test State
G/S/P/G0	7 g PVA solution + 3 g GO/SA ink	0 g	None	Freeze-dried precursor screening
G/S/P/G1	7 g PVA solution + 3 g GO/SA ink	1 g	None	Freeze-dried precursor screening
G/S/P/G2	7 g PVA solution + 3 g GO/SA ink	2 g	None	Selected precursor formulation
G/S/P/G3	7 g PVA solution + 3 g GO/SA ink	3 g	None	Freeze-dried precursor screening
G/S/P/G4	7 g PVA solution + 3 g GO/SA ink	4 g	None	Freeze-dried precursor screening
N-G/S/P/G	G/S/P/G2	2 g already incorporated	11.69 g NaCl per 100 mL initial aqueous treatment medium, 4 h	Solvent-treated comparison gel
F-G/S/P/G	G/S/P/G2	2 g already incorporated	11.69 g NaCl per 100 mL initial water/glycerol treatment medium (1:2, *v*/*v*), 4 h	Low-temperature and sensing gel

**Table 7:** Sample designations and definitions used in this study.

Designation	Definition
PVA	Pure PVA gel
P/G	PVA/glycerol reference gel
G/S/P	GO/SA/PVA gel
G/S/P/G0–G4	GO/SA/PVA precursor containing 0–4 g glycerol per 10 g G/S/P ink
G/S/P/G2	Selected precursor containing 2 g glycerol per 10 g G/S/P ink
N-G/S/P/G	G/S/P/G2 treated in the aqueous-NaCl medium
F-G/S/P/G	G/S/P/G2 treated in the NaCl-containing water/glycerol medium (1:2, *v*/*v*)

## Data Availability

The data supporting the findings of this study are available from the corresponding author upon reasonable request.
